# Cold Starting Temperature Drift Modeling and Compensation of Micro-Accelerometer Based on High-Order Fourier Transform

**DOI:** 10.3390/mi13030413

**Published:** 2022-03-05

**Authors:** Yi Wang, Xinglin Sun, Tiantian Huang, Lingyun Ye, Kaichen Song

**Affiliations:** 1 School of Aeronautics and Astronautics, Zhejiang University, Hangzhou 310007, China; wjhqhf168usst@163.com (Y.W.); kcsong@zju.edu.cn (K.S.); 2 College of Biomedical Engineering & Instrument Science, Zhejiang University, Hangzhou 310007, China; tthuang@zju.edu.cn (T.H.); lyye@zju.edu.cn (L.Y.)

**Keywords:** micro-accelerometer, cold start-up phase, temperature compensation, K-means clustering, symbiotic organisms search

## Abstract

The traditional temperature modeling method is based on the full heating of the accelerometer to achieve thermal balance, which is not suitable for the cold start-up phase of the micro-accelerometer. For decreasing the complex temperature drift of the cold start-up phase, a new temperature compensation method based on a high-order Fourier transform combined model is proposed. The system structure and repeatability test of the micro digital quartz flexible accelerometer are provided at first. Additionally, we analyzed where the complex temperature drift of the cold start-up phase comes from based on the system structure and repeatability test. Secondly, a high-order temperature compensation model combined with K-means clustering and the symbiotic organisms search (SOS) algorithm is established with repeatability test data as training data. To verify the proposed temperature compensation model, a test platform was built to transmit the measured values before and after compensation with the proposed Fourier-related model and the other time-related model, which is also a model aiming at temperature compensation in the cold start-up phase. The experimental results indicate that the proposed method achieves better compensation accuracy compared with the traditional temperature compensation methods and the time-related compensation model. Furthermore, the compensation for the cold start-up phase has no effect on the original accuracy over the whole temperature range. The stability of the accelerometer can be significantly improved to about 30 μg in the start-up phase of different temperatures after compensation.

## 1. Introduction

Quartz flexible accelerometers have been widely used in the fields of national defense and military industry for their properties of small size, quick response, high anti-interference ability, and better long-term stability [1]. Nevertheless, using the traditional analog quartz flexible accelerometer will lead to additional measurement error, which attributes to adding an extra A/D conversion module when in use. With the development of the digital chip, the quartz flexible accelerometer has developed toward digitization. Compared with the analog accelerometer, the digital accelerometer outputs digital signals directly, which avoids the measurement error from the external A/D conversion module. Additionally, the digital chip makes it possible to correct and compensate the closed-loop model parameters in real time. Therefore, the digital accelerometer is easier and more reliable to put into use. In addition, the accuracies of the analog and digital quartz flexible accelerometers are both mainly affected by the temperature, whose influencing mechanism is the comprehensive effect of the external environment temperature and the change in internal materials, electronic components, and structures.

At present, the common temperature compensation methods are hardware and software compensation. The former is derived from the closed-loop system structure of the accelerometer. It usually improves the temperature characteristic through studying the internal structure and material properties to replace heat-resistant material or adding a temperature control circuit [2,3,4,5]. For example, professor Xu’s team has taken measures to reduce the effect of temperature by choosing material with a small temperature coefficient of elastic modulus as the structure of the cantilever beam [6]. Additionally, some researchers install a compensation ring in the torque device to improve the magnetic temperature coefficient of the permanent magnet [7]. The software compensation is mainly based on the mathematical model of a quartz flexible accelerometer. It can realize the real-time temperature compensation by fitting the function expression between the model parameters and temperature. In reference [8,9,10], they calculate the bias and scale factors based on the traditional mathematical expression of the accelerometer with the four-point calibration method. In a different way, refs. [11,12,13,14] take action to transform the traditional mathematical model of the accelerometer with Fourier transform. Then, coefficients can be calculated by accumulating the data acquired all around a circle. Furthermore, in order to improve the nonlinear adaptability of the algorithm, a neural network is adopted to identify the bias and scale factors [15]. Research has shown that it can better fit the model to put the input acceleration and temperature as input variables of the neural network [16]. Compared with the hardware compensation, the software compensation is more suitable for fast and stable starting of the accelerometer for the advantage of avoiding the extra volume and weight and being easier to realize. Nevertheless, the emphasis and difficulty lies in the establishment and identification of a high-precision temperature compensation model.

As the core components of the the inertial navigation system, the precision of the quartz flexible accelerometer directly affects the positioning accuracy of the navigation system. With the rapid development and wide application of strapdown inertial navigation system, it is inevitable to propose higher performance indexes such as starting time, start-up stability, and the maximum drift error to ensure the rapid start and calculation accuracy of the navigation system [17]. Therefore, researching the temperature compensation of the cold start-up phase of the accelerometer is of great significance to enhance the real-time performance and navigation accuracy of the navigation system. However, a common temperature compensation for the accelerometer is conducted with the data collected under the condition of thermal balance but ignoring the output of cold starting. Experimental results indicate the output of the digital quartz flexible accelerometer has characteristics of short-time rapid rise and strong nonlinearity with temperature. At this point, the output is no longer applicable to the compensation model based on temperature and its higher-order terms. To deal with this, many researchers have proposed all kinds of models. The paper [18] proposes a temperature error model related to temperature, its higher-order terms, and its change rate, and the authors utilized a corresponding identification method based on an artificial fish swarm algorithm to verify the stability under different temperature conditions. However, the verification was tested for about 40 h; thus, it is difficult to research the characteristics in the start-up phase. Corresponding to this, reference [19] creatively proposed a time-related temperature compensation model specifically for the cold starting output of the accelerometer. This model approximates the complex output in the cold start-up phase to the response curve related to time and temperature and then utilizes the particle swarm optimization (PSO) algorithm to identify the model parameters. However, the proposed model is too complicated to implement in the processor due to the addition of the time variable and exponential function. In addition, on account of the inevitable difference between the characteristics of the cold start-up output and the system response curve, the time-related model has a limited effect on improving the stability of the accelerometer in the cold starting phase.

Based on this, the focus of this paper is temperature compensation for the cold start-up phase of a micro digital quartz flexible accelerometer. On the basis of sufficient repeatability experiments, a temperature compensation method based on high-order Fourier transform is proposed. According to the experimental test data, the K-means clustering algorithm is utilized to estimate the order of the model. Then, the symbiosis search algorithm with stronger global optimization and local convergence capabilities is used to identify model parameters. Finally, a test platform is built to verify the effect of the temperature compensation. The results show that the comprehensive use of the clustering algorithm and intelligent optimization algorithm greatly improves the efficiency of parameter identification. Furthermore, the proposed model with a simple structure is easy to realize in the processor, which can effectively compensate for the complex temperature drift of the micro quartz flexible accelerometer under the cold starting condition. After compensation, the bias stability significantly improved and remained at about 3 × $10^{-5}$ g.

## 2. Analysis of Cold Start-Up Temperature Characteristics for Micro Digital Quartz Flexible Accelerometer

### 2.1. Basic Model of Digital Quartz Flexible Accelerometer

**Figure 1:**
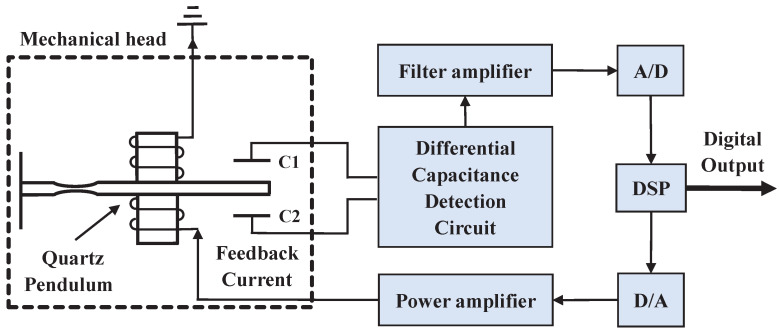
Structure of digital quartz flexible accelerometer.

The micro quartz flexible accelerometer, mainly composed of a mechanical meter and servo circuit, is a kind of closed-loop mechanical pendulum accelerometer. Essentially, it is a mass-spring-damping system. Compared with a traditional analog quartz flexible accelerometer, the digital one appends the AD conversion module, digital signal processor, and DA drive module so as to form a complete digital closed-loop system. The structure of the digital quartz flexible accelerometer is shown in Figure 1.

When the acceleration input changes, the pendulum generates slight displacement, and with the same time differential, the capacitance formed by the magnetic steel on both sides of the pendulum increases on one side and decreases on another side. After the demodulation of the differential capacitance detection circuit and the amplification of the filter amplifier, the measured differential capacitance signal is finally converted to a digital signal through the AD conversion module and then sent to the digital signal processor. Next, the digital output calculated by the controller is transmitted to the DA drive module in order to generate electromagnetic torque through the power amplifier to make the pendulum keep in balance. In a closed-loop system, the feedback current is proportional to the measured acceleration so that it can be acquired as the measured acceleration to output the digital value by the digital signal processor (DSP) directly. The relationship between feedback current and input acceleration is expressed as [20]:

(1) $$i=\frac{m}{Bl}a+\frac{b}{Bl}=Ka+K_{0}$$

where *i* is the feedback current with unit A, *m* represents the quality of the quartz pendulum component whose unit is Kg, *a* is the input acceleration with unit m/s${}^{2}$, *B* is the magnetic induction intensity between the working air gap with unit T, *l* is the length of the torquer coil whose unit is m, *b* is the error term of the feedback force with unit N, *K* is the scale factor, and *K*_0_ is the partial value.

### 2.2. The Temperature Characteristic for the Cold Start-Up Phase

**Figure 2:**
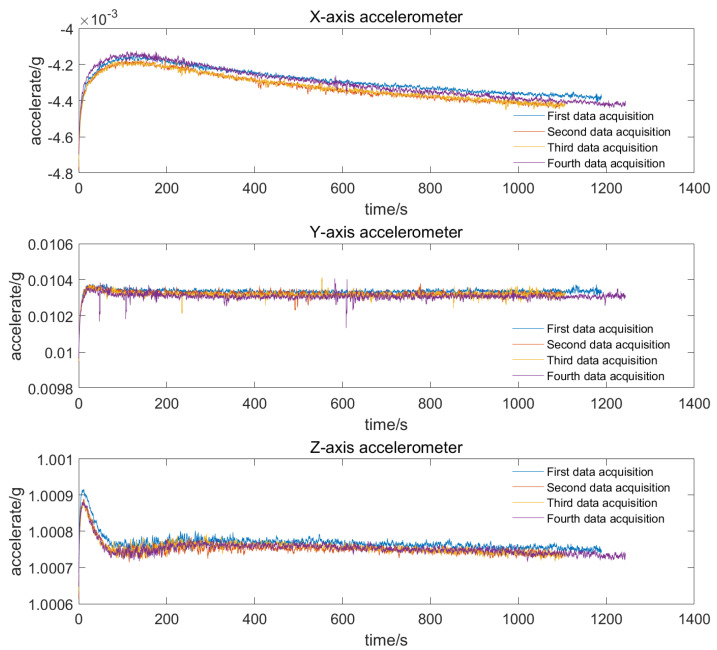
The experimental results for cold start-up phase of three-axes accelerometer.

The test object is the digital three-axis integrated accelerometer developed by our laboratory. For fully studying the output characteristics during the cold start-up phase, experiments were conducted repeatedly under vibration isolation, noise reduction, and room temperature (27 ± 0.5 °C) environmental conditions. We turned on the power and ran the test for half an hour, and then turned it off to cool down for an hour. The corresponding experimental results for the three axes are shown in Figure 2.

In order to better observe the output characteristics in the cold start-up phase, the figure enlarges the curve in the first 100 s. The following can be summarized from the experimental results:

(1) Different accelerometers display various characteristics for the cold start-up phase;
(2) Different accelerometers for the cold start-up phase generally show a rapid rise within a short time and gradually slow down until stabilization;
(3) The output of different accelerometers has strong nonlinearity with temperature for the cold start-up phase;
(4) The temperature drift repeats well under identical environmental conditions, which lays the foundation for temperature compensation.

On the basis of the above characteristics, it is necessary for the proposed temperature compensation model to have strong nonlinear adaptability and for the corresponding identification algorithm to have the ability of global search and local fast convergence.

### 2.3. Mechanism Analysis of Complex Temperature Drift for Cold Start-Up Phase

According to the structure of the accelerometer shown in Figure 1, the source of temperature drift is mainly divided into two parts: mechanical components and the circuit of closed-loop control.

(1) Due to the thermal effect of the feedback current, the complex drift is caused by the thermal deformation of the structure, the change in linear expansion coefficient for the quartz pendulum, the temperature coefficient of the magnetic steel, and the coil. These errors are derived from elastic elements, differential capacitors and magnetic circuits of mechanical components;
(2) Considering the perspective of circuit, functional chips such as amplifier chips have the characteristic of start-up drift. Moreover, basic components of the circuit such as resistors and capacitors shift more when cold starting.

In summary, the complex temperature drift of the quartz flexible accelerometer in the cold start-up phase is the synthetic influence of the start-up drift characteristics of the components and the temperature effect of the physical coefficients. The output presents the characteristics of a short-time rise and strong nonlinearity with temperature.

## 3. Temperature Compensation Model of Micro Digital Quartz Flexible Accelerometer

### 3.1. Traditional Temperature Compensation Model

In the gravitational field, the mathematical model of the quartz flexible accelerometer is usually expressed as [8,10,13,21]:

(2) $$U=K_{0}+K_{1}a+K_{2}a^{2}+K_{c}a+\varepsilon a^{{}^{\prime }}$$

where *U* is the output of the accelerometer, *K${}_{0}$*, *K${}_{1}$*, *K${}_{2}$*, and *K${}_{c}$* are the partial value, the scale factor, the two-order nonlinear coefficient, and the cross-coupling coefficient, respectively, ε is the installation error term, *a* is the input acceleration, and *a${}^{{}^{\prime }}$* is the component in the vertical direction of the input acceleration.

Practical application shows that the temperature change mainly affects zero offset *K${}_{0}$* and scale factor *K${}_{1}$*, and the remaining terms can be approximated as decimal terms. Therefore, the compensation of zero bias and scale factor should be considered emphatically. To achieve the purpose of real-time temperature compensation, this is the most common way to establish a compensation model connected to temperature with zero bias and scale factor. Furthermore, the temperature compensation model related to temperature, its high-order terms, and its change rate are expressed as follows [10,11,13,18]:

(3) $$\left\{\begin{array}{c} K_{0}\left(T,\Delta T\right)=K_{00}+K_{01}T+K_{02}T^{2}+\ldots +K_{0n}T^{2}+K_{0n+1}\Delta T+\ldots +K_{0n+m}{\left(\Delta T\right)}^{m}\\ K_{1}\left(T,\Delta T\right)=K_{10}+K_{11}T+K_{12}T^{2}+\ldots +K_{1n}T^{2}+K_{1n+1}\Delta T+\ldots +K_{1n+m}{\left(\Delta T\right)}^{m} \end{array}\right.$$

where *T* is the temperature, ΔT is the temperature gradient, *K${}_{00}$*, ⋯, *K${}_{0n+m}$*, *K${}_{10}$*, ⋯, *K${}_{1n+m}$* are the coefficients of the temperature compensation model. In general applications, this model can effectively decrease the temperature drift of zero bias and scale factor. However, it can be seen from Formula (3) that the output of the model is still monotonic with the temperature, which means the model has a poor applicability in the cold start-up phase whose output presents a complex change where the temperature rises monotonously.

### 3.2. Temperature Compensation Model Based on High-Order Fourier Transform

This model is inspired by the trigonometric function expression form of Fourier transform which is commonly used in the field of signal processing. The Fourier transform reveals to us an important law that any periodic function can be regarded as the superposition of sine waves with different amplitudes and phases. Therefore, to compensate the short-time nonlinear output for the cold start-up phase, a new type of Fourier transform-based temperature compensation model is proposed. The mathematical expression of the model is shown as Equation (4) [22]:

(4) $$\Delta U\left(T\right)=a_{0}+\sum_{i=1}^{n}\left[a_{i}cos\left(iwT\right)+b_{i}sin\left(iwT\right)\right]$$

where ΔU(T) is the output error of the accelerometer, *a${}_{0}$, a${}_{i}$, b${}_{i}$, w* are the corresponding parameters of the model, and n is the order of the Fourier transform.

There is strong nonlinear adaptability for the Fourier transform itself, and the higher the order, the stronger the nonlinear fitting ability. Hence, theoretically, the model can better fit the complex temperature characteristics for the accelerometer’s cold start-up phase. The emphasis is on the calculation of the order of the Fourier transform and other model parameters. Therefore, to improve the efficiency of the compensation, research into clustering algorithms for data feature extraction and intelligent optimization algorithms to optimize model parameters has been undertaken.

## 4. Modeling Method for Temperature Compensation Model

### 4.1. K-Means Clustering Algorithm Based on SSE

The K-means clustering algorithm is a kind of unsupervised learning algorithm based on partition, usually using Euclidean distance to measure the similarity between data [23]. The algorithm assigns all data points to the cluster with the nearest cluster center point by specifying the number of cluster centers and the initial cluster center point. After the allocation, it calculates the average value of all clusters and allocates the data again until the clustering centers move within a small limited range.

In the temperature compensation of the cold start-up phase on the basis of the high-order Fourier transform model, the order is an unknown parameter, which increases the identification complexity for the parameter optimization. Therefore, the K-means clustering algorithm is considered to estimate the order of Fourier transform. The sum of squares errors (SSE) for the overall data is regarded as the evaluation index. With the number of clusters increasing, the gradient of SSE will decrease sharply. Therefore, we draw the correlation curve between the K value and corresponding SSE, and the K value corresponding to the maximum change rate of SSE is the optimal number of clustering centers. The method is also called the elbow method, and the SSE for the overall data is calculated with the following formula [24]:

(5) $$SSE=\sum_{i=1}^{K}\sum_{X\in C_{i}}{\left|dis(X,C_{i})\right|}^{2}$$

where *X* is the data set; *K* is the number of clusters; and *C${}_{i}$* is the cluster center.

According to the improved K-means clustering algorithm, the output of the accelerometer in cold start-up stage is to be analyzed to provide a preliminary estimate the order of the temperature compensation model based on Fourier transform. The clustering results of the three axes for the optimal K value are 4 for the *X*-axis, 5 for the *Y*-axis and 6 for the *Z*-axis.

### 4.2. Symbiotic Organisms Search Intelligent Algorithm

The symbiotic organisms search intelligent algorithm (SOS) is a kind of swarm intelligence optimization algorithm that is used to simulate the ubiquitous interaction behaviors between individuals in nature. The symbiosis relationships are mainly divided into mutualism, commensalism, and parasitism, which is exactly the principle used to enhance the environmental adaptability between species in the nature ecosystem. Compared with another commonly used swarm intelligence optimization algorithm called particle swarm optimization algorithm (PSO), SOS equips three different population screening mechanisms, while PSO only relies on speed and location updates. So, in theory, SOS is better than PSO in global optimization and local convergence [25]. Similar to other swarm intelligence optimization algorithms, SOS filters optimal individuals through population iteration. The main steps of SOS are described as follows:

(a) Mutualism
  Mutualism is the most common symbiotic relation in nature, which means that both participants gain benefits from the symbiotic interaction. In the mutualism of the algorithm, for any individual *X${}_{i}$*, *X${}_{j}$* is randomly chosen to interact with and remain different from *X${}_{i}$*. The individuals’ update formula is described in Equation (6):
  (6) $$\left\{\begin{array}{c} X_{inew}=X_{i}+rand*\left(X_{best}-MV*BF_{1}\right)\\ X_{jnew}=X_{j}+rand*\left(X_{best}-MV*BF_{2}\right)\\ MV=\frac{X_{i}+X_{j}}{2} \end{array}\right.$$
  in Equation (6), rand is a random number between 0 and 1, *BF${}_{1}$* and *BF${}_{2}$* represent beneficial factors with the value of either 1 or 2, *X${}_{inew}$* and *X${}_{jnew}$* are corresponding individuals after update, and *X${}_{best}$* donates the greatest individual adaptation; the last parameter *MV* is the average of *X${}_{i}$* and *X${}_{j}$*, which indicates the common characteristics between *X${}_{i}$* and *X${}_{j}$*. Therefore, (*X${}_{best}$− MV ∗ BF*) signifies that *X${}_{i}$* and *X${}_{j}$* work together to get closer to *X${}_{best}$*. According to the formula (6), individuals of the initial population all need to be updated and then compared with the previous ones. If it is better than the former one, *X${}_{i}$* and *X${}_{j}$* would be replaced with *X${}_{inew}$* and *X${}_{jnew}$*.
(b) Commensalism
  Commensalism is a kind of symbiotic relationship among two organisms where one organism could benefit while the other receives nothing. Just as with the mutualism phase, we select a random organism *X_j_* to interact with organism *X_i_* and update them in accordance with Formula (7):
  (7) $$X_{inew}=X_{i}+rand*\left(X_{best}-X_{j}\right)$$
  where the definition of related variables is the same as Equation (6). So, (*X${}_{best}$−X${}_{j}$*) reflects the assistance *X${}_{j}$* provides to *X${}_{i}$* to improve its adaption level. At last, we choose the better one from *X${}_{i}$* and *X${}_{inew}$*.
(c) Parasitism
  Parasitism is a kind of elimination mechanism for organisms in a natural form in which one organism benefits and at the same time has an adverse effect on the other organism. The relationship is described as follows:
  (8) $$\left\{\begin{array}{c} X_{inew}=X_{i}\\ d=ceil(rand*D)\\ X_{inew}\left(d\right)=X_{i}\left(d\right)+rand*\left(X_{dmax}-X_{dmin}\right) \end{array}\right.$$
  where *D* is the dimension of each individual; *ceil* is a function to obtain the integer; and *d* represents any integral dimension ranging from 1 to *D*; *X${}_{dmax}$* and *X${}_{dmin}$* indicate the maximum and minimum value of range in the *d* dimension. If the value of *X${}_{inew}$* is larger than that of *X${}_{i}$*, then it will eliminate *X${}_{i}$* and replace its position in the population.

## 5. Identification and Compensation of Temperature Compensation Model

### 5.1. Experimental Design of Temperature Compensation

**Figure 3:**
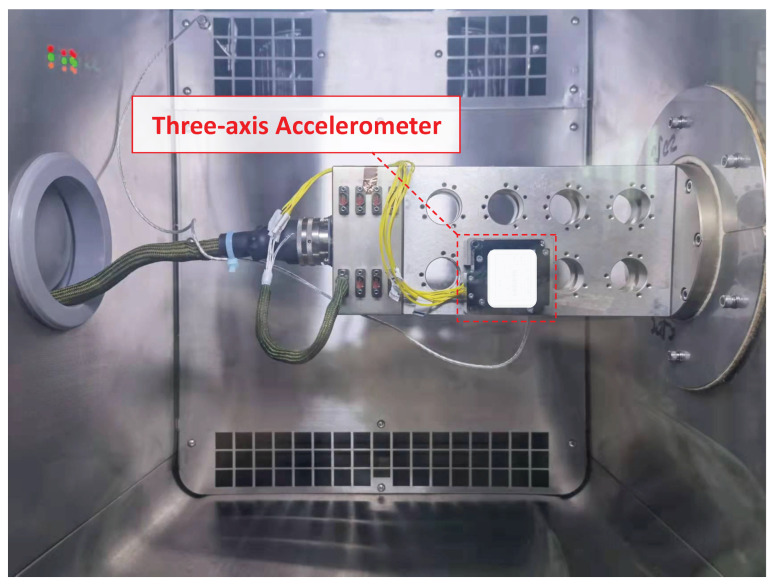
Experimental platform of accelerometer for temperature compensation.

It can be concluded from the experimental results that the characteristics of the cold start-up phase are not suitable for the traditional temperature compensation model, while the output gradually stabilizes and fits the traditional temperature compensation model after the accelerometer reaches thermal equilibrium. Therefore, considering piecewise compensation, it would be more useful to compensate the cold start-up phase with a high-order Fourier transform model and to decrease output error after reaching thermal balance with a traditional compensation model. With that in mind, the overall temperature compensation experiment of the micro-accelerometer is mainly divided into two parts, traditional temperature compensation in thermal equilibrium and compensation with a new model for the cold start-up phase. As shown in Figure 3, the experimental platform includes a high-precision controllable thermostat, rotary table, power supply, industrial computer, and matched software for data acquisition.

The experiment object is a micro three-axis closed-loop quartz flexible accelerometer independently developed by the laboratory with the advantages of outputting measured acceleration in real time through 422 bus and display software for programming temperature compensation model parameters. Since this article focus more on the temperature compensation in the cold start-up phase, the traditional compensation for stable output after thermal equilibrium will not be described too much. The specific experimental steps for temperature compensation are designed as follows:

Step 1: Put the digital accelerometer in a high-precision thermostat to fully heat it until it has a stable output. Then, set the range of temperature for the thermostat to −40 to 60 °C and keep the change rate of temperature change at 1 °C/min. At different stable temperature points, four-point roll experiments are conducted to obtain zero offset and scale factor change with temperature. After that, it is usually used to obtain parameters of the traditional compensation model.

Step 2: Control the thermostat to room temperature and power it off until the accelerometer cools sufficiently. Then, turn on power to conduct the experiment for half an hour to acquire a set of valid data and then turn the power off. Repeat the experiment to verify the characteristics of the accelerometer for the cold start-up phase to ensure the feasibility of the subsequent temperature compensation.

Step 3: On the basis of step 2, three sets of data are selected from each of the three axes as training data to identify the parameters of the new temperature compensation model through the proposed K-means clustering algorithm and SOS intelligent optimization algorithm.

Step 4: Program the identified parameters to the microprocessor and modify the program to output the measured data before and after temperature compensation at the same time. Finally, display the collected data to verify the effectiveness of temperature compensation.

### 5.2. Identification of the Temperature Compensation Model

**Figure 4:**
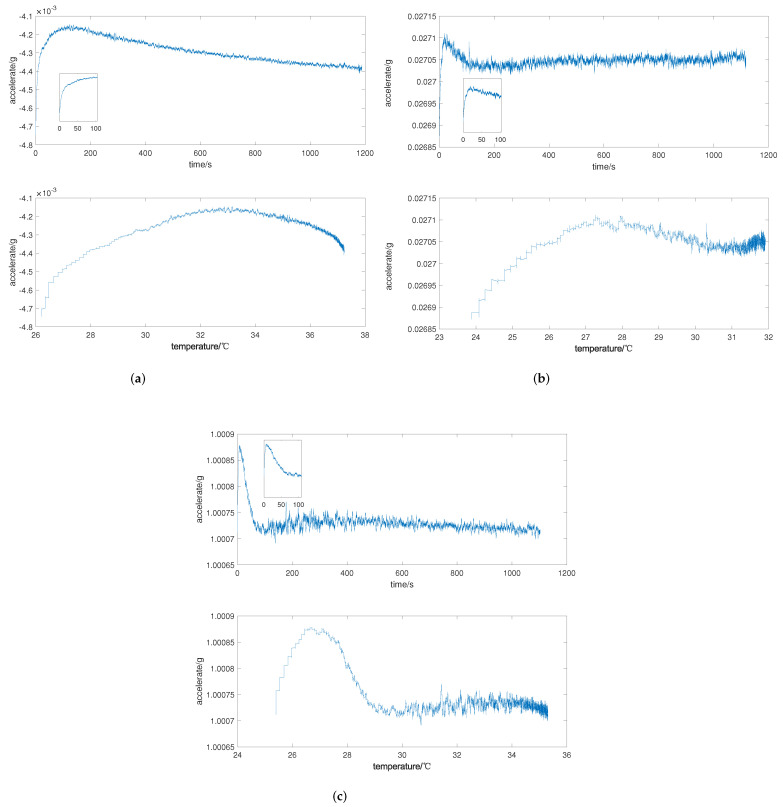
Time- and temperature-domain diagram of three axes for cold start-up phase: (**a**) *X*-axis accelerometer; (**b**) *Y*-axis accelerometer; (**c**) *Z*-axis accelerometer.

According to experimental steps, take the tested data of the accelerometer for the cold start-up phase as training data. To better observe and identify the output characteristics, this is included in Figure 4, which shows the time-domain diagram of the *X*, *Y*, and *Z* axes and the corresponding output curves’ change with temperature. It can be concluded from the figure that the data on temperature for the cold-starting phase are strongly nonlinear, and that the characteristics in the cold start-up phase differ in different accelerometers.

**Figure 5:**
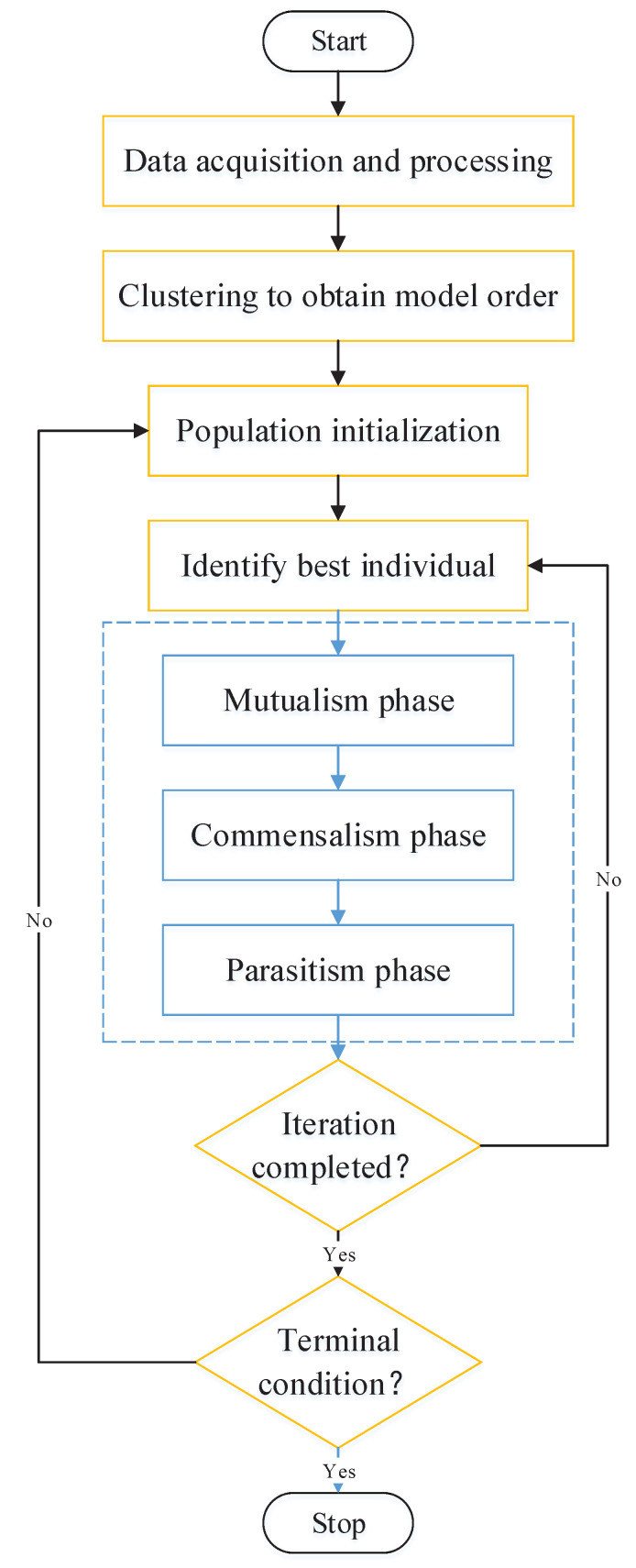
The identification flowchart of K-means–SOS–Fourier method for temperature compensation.

In order to better fit the curve of the temperature characteristic in the cold start-up phase, this paper proposes a method called K-means–SOS–Fourier to estimate the model order with the K-means clustering algorithm and then identify the parameters of the model through the SOS intelligent algorithm. It should be noted that the corresponding data pre-processing is required before parameter identification of the temperature compensation model. The output data are firstly processed with the Kalman filtering algorithm in the processor to avoid data missing. Secondly, data acquisition has been tested for a long time before temperature compensation to ensure the stability of accelerometer output. Simultaneously, the experiments of temperature compensation were conducted in the environment of vibration isolation and noise reduction. The noise contained in the output data is mainly composed of environmental white noise. Therefore, after data preprocessing with the Kalman filter, it is still necessary to utilize the sliding filtering algorithm to filter out environmental noise. Then, the filtered data can be considered as the actual acceleration output that contained temperature drift. Furthermore, data missing is the key to improve the reliability of high-precision sensors. Bayesian estimation is an efficient method to reconstruct missing data for telling us how to calculate the reliability of the final estimated value when the reliability of the observation value and control command are known [26,27,28]. The Kalman filter is a special application of Bayesian filtering in which the credibility model of the observed value and control are in line with normal distribution [29]. The measured noise and observed noise in accelerometers are in accordance with normal distribution. Therefore, the data preprocessing algorithm that combined Kalman filter and sliding filter can effectively ensure the stability and accuracy of the accelerometer output [30,31]. In addition, there are two different terminal conditions for the whole identification process. One is to reach the specified number of cycles and the other is to meet the error accuracy. The flowchart for model identification is shown in Figure 5.

**Figure 6:**
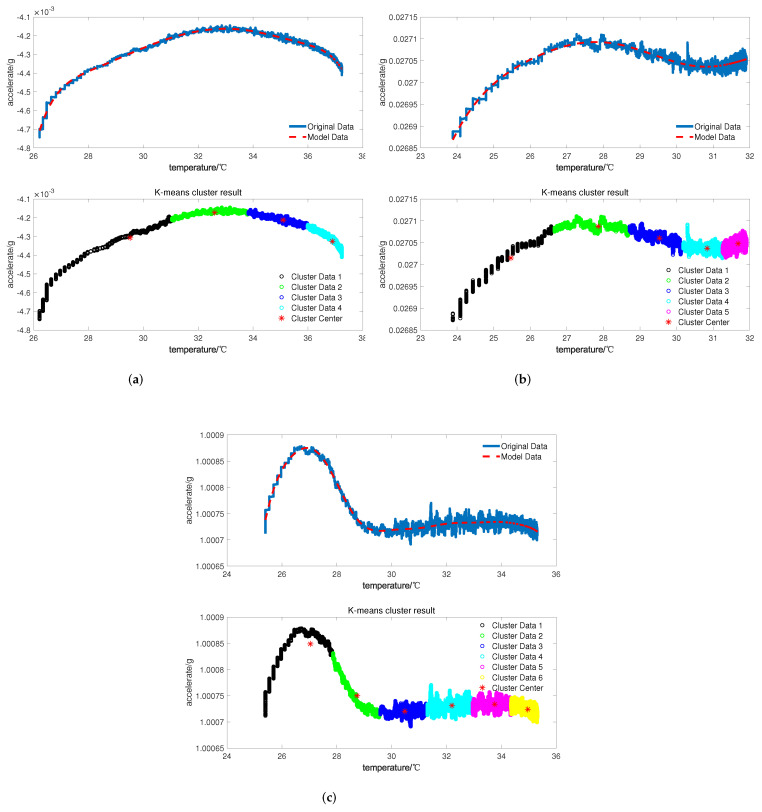
Identification diagram of the model for the three-axial accelerometer: (**a**) *X*-axis accelerometer; (**b**) *Y*-axis accelerometer; (**c**) *Z*-axis accelerometer.

The acquired data are identified according to the designed temperature compensation model, and the calculated results of the clustering algorithm and intelligent algorithm are shown in Figure 6.

It can be seen from Figure 6 that the temperature characteristic curve of the *X*-axis with weak nonlinearity corresponds to a small number of cluster centers, while the curve of the *Z*-axis has strong nonlinearity so that the corresponding number is bigger. This further proves that the elbow method can accurately estimate the order of the proposed temperature compensation model according to the nonlinearity of the temperature–drift curve.

### 5.3. Verification of Temperature Compensation Model

To verify the feasibility and efficiency of the proposed temperature compensation model, data for the repeatability test were utilized to identify the time-related model proposed in [19] and the Fourier-related model for the cold start-up phase. After that, we program the model parameters and set the processor to output the data before and after temperature compensation with two different models. Then, tests for the cold start-up phase are performed and display the results, including original data marked with a blue line, model-fitted data labeled with a red line, and the results after compensation marked with a yellow line.

**Figure 7:**
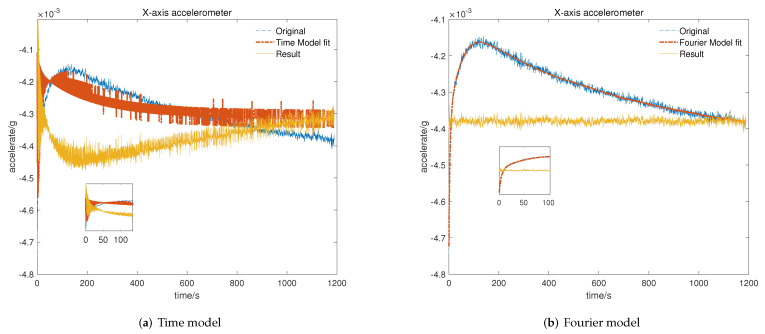
Result of temperature compensation for *X*-axis accelerometer: (**a**) compensation with time-related model; (**b**) compensation with Fourier-related model.

**Figure 8:**
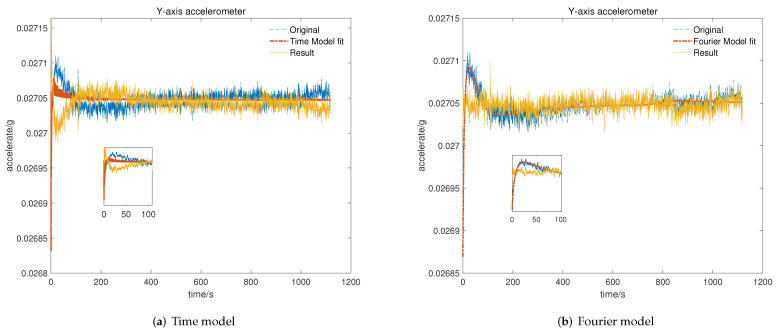
Result of temperature compensation for *Y*-axis accelerometer: (**a**) compensation with time-related model; (**b**) compensation with Fourier-related model.

**Figure 9:**
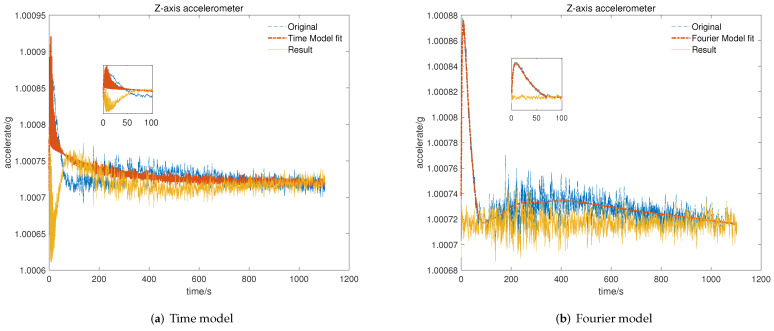
Result of temperature compensation for *Z*-axis accelerometer: (**a**) compensation with time-related model; (**b**) compensation with Fourier-related model.

As shown in Figure 7, Figure 8 and Figure 9, the time-related model on the basis of impulse response has poor ability to fit the short-term rapid rising curve, which is attributed to differences in output characteristics between different axial accelerometers and the impulse response. In contrast, the Fourier-related model is based on the temperature characteristic curve of the cold start-up phase, and the nonlinear fitting ability of the model increases with the increase in order, which makes it feasible to apply it to temperature compensation with different complex output characteristics. We can conclude that the output of the accelerometer in the cold start-up phase after temperature compensation with the proposed K-means–SOS–Fourier method is more stable compared to the compensation with the time-related model. In Table 1, the stability and the maximum drift error before and after compensation with two different models are shown.

It can be seen from Table 1 that the output characteristics of the micro-accelerometer in the cold start-up phase are improved after temperature compensation with two different models. Nevertheless, compared with the time-related model proposed in [19], the temperature compensation model on the basis of high-order Fourier transform has a better effect on compensating the output drift of the accelerometer in the cold starting section. After compensation with the time-related model, since the time characteristics of the output curve in the cold start-up phase are not exactly same as those of the impulse response curve, the compensation for the initial rapid rising section is so poor that there is only slight improvement in the stability and the maximum drift error compared with the original data. In comparison, due to the strong ability to fit the complex characteristic curve, the Fourier-related model can effectively compensate the short-term and fast output of the starting section. Thus, the stability of the cold start-up output is greatly improved to be kept within 1 × $10^{-5}$ g and the maximum drift error is limited to 5 × $10^{-5}$ g with the temperature compensation of the Fourier-related model. This further verifies that the proposed model is more applicable and possesses better theoretical output stability and maximum output drift performance after compensation than the time-related model.

**Figure 10:**
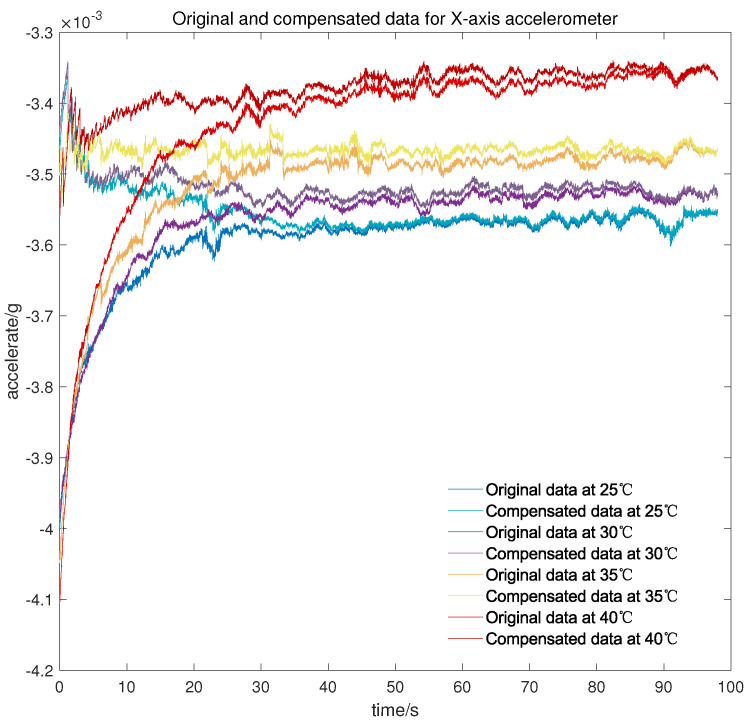
Original and compensated data for *X*-axis accelerometer at different temperature points.

**Figure 11:**
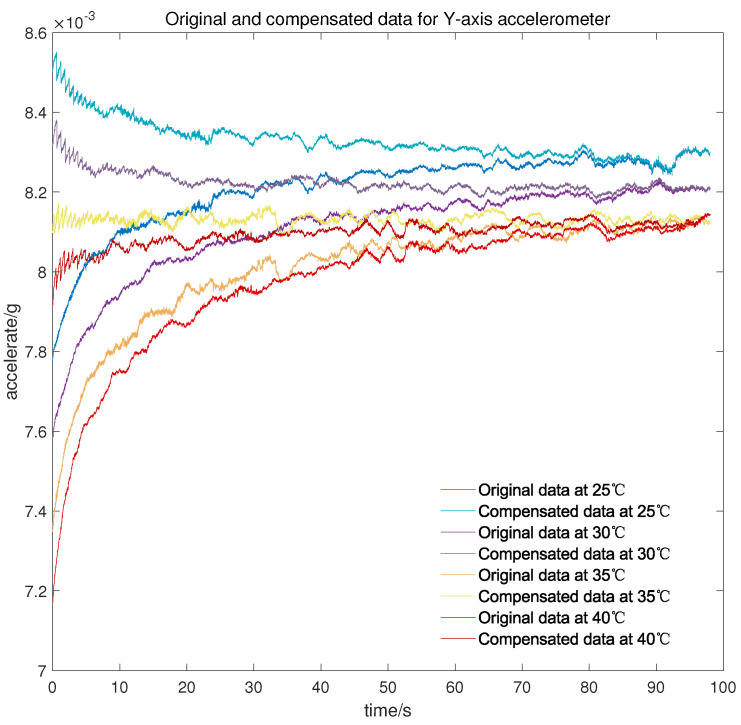
Original and compensated data for *Y*-axis accelerometer at different temperature points.

**Figure 12:**
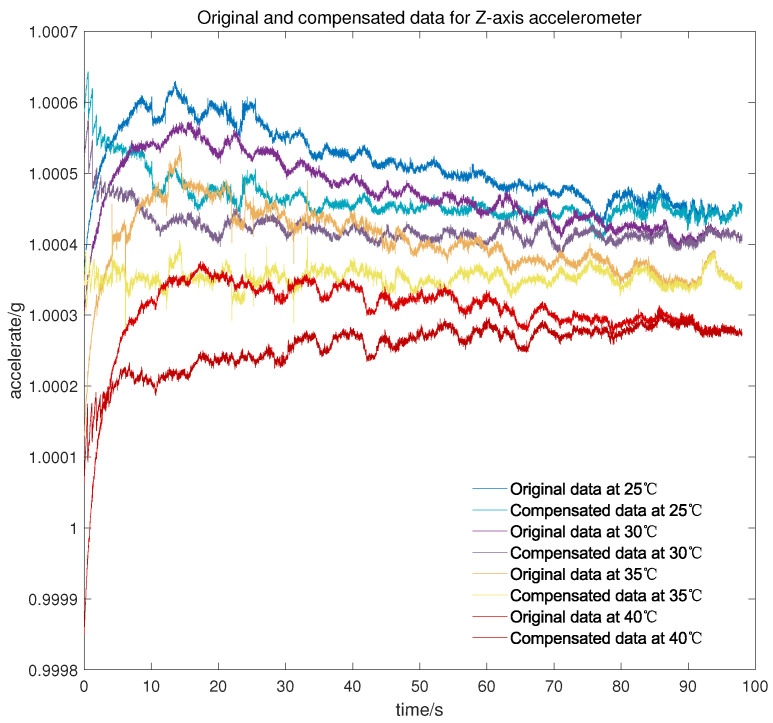
Original and compensated data for *Z*-axis accelerometer at different temperature points.

Meanwhile, to verify the applicability of the model in the cold start-up phase at different temperature points, another micro three-axis accelerometer is adopted to conduct the cold start-up tests for about 100 s at 25 °C, 30 °C, 35 °C, and 40 °C, respectively. As can be seen from Figure 10, Figure 11 and Figure 12, there is still a small amount of residual drift after the compensation, which is attributed to slight differences in the output characteristic in the cold start-up phase at different temperature points. Furthermore, the short-term output of the accelerometer after compensation is serrated because the proposed temperature compensation model is continuous in the temperature domain and the output changes greatly within the same measured temperature. There are two methods for the stability statistics of the accelerometer. One is on the basis of the output data over a continuous period of time, and the other is to count the mean value of the output data at different temperature points. The proposed model is mainly used to improve the stability under the first statistic, but it is also worth listing the mean value before and after compensation.

It can be concluded from Table 2 that after the compensation of the proposed model, the cold start-up output of the micro-accelerometer at different temperature points is maintained at about 3 × $10^{-5}$ g, which is greatly improved compared with the accuracy before compensation. Simultaneously, the data volume of complex output in the cold starting section is less than that of the overall test output data so that the compensation for the cold start-up phase has less effect on temperature constancy of the accelerometer, and the accelerometer itself has good performance in changeable temperature according to the value shown in Table 3.

In conclusion, all tests provide strong evidence for the applicability of the compensation model in the cold start-up phase.

## 6. Conclusions

In this paper, research and experiments have been carried out on the complex temperature characteristics of the micro digital quartz flexible accelerometer for the cold start-up phase. After that, a temperature compensation model based on the high-order Fourier transform triangle expression is proposed. Meanwhile, in order to improve the identification efficiency of the temperature compensation model, an improved K-means clustering algorithm combined with the symbiotic organisms search (SOS) intelligent algorithm is proposed. To verify the effectiveness of the proposed temperature compensation method, an experimental test platform is built. After traditional temperature compensation, the proposed K-means–SOS–Fourier method is utilized to perform specific temperature compensation for the start-up phase. The experimental results show that the proposed temperature compensation method, K-means–SOS–Fourier, can effectively reduce the complex drift derived from temperature change in the cold start-up phase. Specifically, the stability of the cold start-up output can be maintained at about 3 × $10^{-5}$ g under different temperature conditions, and the maximum drift error can be kept within 5 × $10^{-5}$ g theoretically, which are good enough measures to meet the requirements of a fast and stable start for a high-precision inertial navigation system. Additionally, after practical verification, the proposed temperature compensation model based on high-order Fourier transform for the cold start-up phase has the following advantages over the time-related compensation model proposed in the literature [19]:

(a) The proposed model possesses a simple structure, and its basic expression can be described as the accumulation of trigonometric functions, which makes it easier to be programmed in the processor than the time-related temperature compensation model for containing exponential terms;
(b) The time-related model contains time variables and exponential functions so that the resource consumption in the processor gradually increases with time. The proposed model is only related to temperature when model parameters are determined, which greatly increases the engineering practicability of the model;
(c) The particle swarm optimization (PSO) used in the time-related model has poor convergence ability in the case of multivariate large-scale optimization, so as a result, it has large residuals of model fitting and low efficiency for model identification. In contrast, the proposed K-means–SOS–Fourier method utilizes an improved clustering algorithm to estimate the model order and then identify model parameters with the symbiotic organisms search (SOS) intelligent algorithm whose global optimization and local convergence capabilities are better than PSO. All the measures have been proved to improve the accuracy and efficiency of model identification effectively;
(d) The actual output of the accelerometer in the cold start-up phase is complex and diverse so that it is impossible for the output to be completely consistent with the characteristics of the response curve. Therefore, there will be still a large residual after temperature compensation with the time-related model. However, the proposed model, on the basis of high-order Fourier transform, can adapt to stronger nonlinear curves with the increase in the model orders, which means it is more practical in actual engineering applications.

In summary, the proposed temperature compensation model is more valuable to engineering applications. Nevertheless, the proposed model also has some limitations. Firstly, the proposed temperature compensation model is only effective for the output of the micro-accelerometer in the cold start-up phase. After reaching thermal equilibrium, the output would be suitable for the traditional compensation model. Secondly, the compensation model is aimed at temperature compensation under specific thermal and environmental conditions. Additionally, the model needs to be re-identified once the surrounding thermal environment changes. Further research will focus on combining temperature characteristics in the cold start-up phase with temperature characteristics after thermal equilibrium. Moreover, we will investigate adding more time-related parameters into the model to improve its adaptability under different temperature environments. 

## Figures and Tables

**Table 1:** Indexes of accelerometer in cold start-up stage before and after temperature compensation.

	Before Temperature Compensation		After Temperature Compensation with Time-Related Model		After Temperature Compensation with Fourier-Related Model	
	**Stability**	**Maximum Drift Error**	**Stability**	**Maximum Drift Error**	**Stability**	**Maximum Drift Error**
*X*-axis ($10^{-6}$ g)	70.432	597.76	59.659	365.98	6.0296	43.461
*Y*-axis ($10^{-6}$ g)	13.072	238.19	12.958	121.43	6.9997	40.058
*Z*-axis ($10^{-6}$ g)	22.362	187.17	18.349	87.5	6.5028	37.164

**Table 2:** Stability of accelerometer in cold start-up stage at different temperature points for three-axis accelerometer.

	*X*-Axis Accelerometer		*Y*-Axis Accelerometer		*Z*-Axis Accelerometer	
	**Before**	**After Compensation**	**Before**	**After Compensation**	**Before**	**After Compensation**
25 °C ($10^{-6}$ g)	64.928	29.746	92.636	47.278	51.230	32.480
30 °C ($10^{-6}$ g)	74.020	22.035	118.01	25.301	48.679	22.640
35 °C ($10^{-6}$ g)	85.229	9.5608	138.57	12.670	47.890	12.032
40 °C ($10^{-6}$ g)	113.02	28.493	168.68	29.941	54.658	31.651

**Table 3:** Mean value of accelerometer at different temperature points for three-axis accelerometer.

	*X*-Axis Accelerometer		*Y*-Axis Accelerometer		*Z*-Axis Accelerometer	
	**Before**	**After Compensation**	**Before**	**After Compensation**	**Before**	**After Compensation**
25 °C	−0.003596	−0.003541	0.008209	0.008329	1.000512	1.000463
30 °C	−0.003566	−0.003517	0.008105	0.008223	1.000472	1.000422
35 °C	−0.003516	−0.003466	0.008008	0.008131	1.000403	1.000352
40 °C	−0.003429	−0.003378	0.007974	0.008096	1.000305	1.000260

## Data Availability

The data presented in this study are available from the corresponding author, Y.W., upon reasonable request.
